# Fasting and Feeding Signals Control the Oscillatory Expression of *Angptl8* to Modulate Lipid Metabolism

**DOI:** 10.1038/srep36926

**Published:** 2016-11-15

**Authors:** Fabin Dang, Rong Wu, Pengfei Wang, Yuting Wu, Md. Shofiul Azam, Qian Xu, Yaqiong Chen, Yi Liu

**Affiliations:** 1 Key Laboratory of Nutrition and Metabolism, Institute for Nutritional Sciences, Shanghai Institutes for Biological Sciences, University of Chinese Academy of Sciences, Chinese Academy of Sciences, 320 Yueyang Road Shanghai, 200031, China; 2 Department of Endocrinology, The First Affiliated Hospital of Harbin Medical University, No. 23 Youzheng Street, NanGang District, Harbin, 150001, China

## Abstract

Emerging evidence implies a key role of angiopoietin-like protein 8 (*Angptl8*) in the metabolic transition between fasting and feeding, whereas much less is known about the mechanism of its own expression. Here we show that hepatic *Angptl8* is rhythmically expressed, which involving the liver X receptor alpha (LXRα) and glucocorticoid receptor (GR) modulation during feeding and fasting periods, respectively. In addition, *Angptl8* mRNA is very unstable, which contributes to the nature of its daily rhythmicity by rapidly responding to fasting/feeding transition. To explore its pathological function in dexamethasone (DEX)-induced fatty liver, we reversed its suppression by glucocorticoids through adenoviral delivery of *Angptl8* gene in mouse liver. Surprisingly, hepatic overexpression of Angptl8 dramatically elevated plasma triglyceride (TG) and non-esterified fatty acid (NEFA) levels in DEX-treated mice, suggesting a metabolic interaction between Angptl8 and glucocorticoid signaling. Moreover, intracellular hepatic Angptl8 is implicated in the regulation of lipid homeostasis by the experiments with ectopic expression of a nonsecreted Angptl8 mutant (Δ25-Angptl8). Altogether, our data demonstrate the molecular mechanism of the diurnal rhythm of *Angptl8* expression regulated by glucocorticoid signaling and LXRα pathway, and provide new evidence to understand the role of Angptl8 in maintaining plasma TG homeostasis.

Hypertriglyceridemia is one of major risk factors of nonalcoholic fatty liver (NAFL), and elevated serum triglycerides (TG) commonly associates with insulin resistance and represent a valuable clinical marker of the metabolic syndrome^1^,^2^. Plasma TG concentrations are mainly determined by the balance between their production and clearance. The former involves the synthesis of very low density lipoprotein triglyceride (VLDL-TG) and chylomicron triglyceride (chylomicron-TG) in the liver and small intestine^3^,^4^, respectively, while the latter involves lipoprotein lipase (LPL)-mediated lipolysis of VLDL-TG^5^.

Angiopoietin-like proteins (Angptls) are a family of proteins structurally similar to the angiopoietins. Angptls have emerged as crucial regulators of circulating TG levels and thus have been expected as potential targets for metabolic syndrome therapy in recent years^6^,^7^. For instance, Angptl3 and Angptl4 have been reported to inhibit LPL activity to impair TG clearance^8^,^9^,^10^. Several studies with *Angptl8*-deficient and/or Angptl8-overexpressing mice have revealed that Angptl8, similar to Angptl3 and 4, plays an important role in modulating circulating TG clearance via the inhibition of LPL activity^11^,^12^,^13^. Of note, overexpression of Angptl3 alone does not alter the levels of circulating TG, whereas co-expressing it with Angptl8 results in hypertriglyceridemia in mice^11^. On the other hand, ectopic expression of Angptl8 in *Angptl3*-null mice has also little effect on plasma TG levels^11^. These results indicate that Angptl3 and Angptl8 have to function together *in vivo*.

Previous studies have shown that *Angptl8* is dramatically suppressed by fasting and significantly induced by refeeding in mice^12^,^14^,^15^. Although many efforts have been taken^12^,^15^,^16^,^17^, the regulatory mechanisms of *Angptl8* gene expression remain inconclusive. Here we show that the liver X receptor alpha (LXRα) and glucocorticoid receptor (GR) are involved in mediating the induction and suppression of *Angptl8* expression in mouse liver during feeding and fasting periods, respectively, which combines with the very short half-life of *Angptl8* mRNA to result in the circadian pattern of its expression. Additionally, results from the experiments of overexpressing Angptl8 in mouse liver suggest that Angptl8 affects hepatic expression of genes involved in non-esterified fatty acid (NEFA) uptake, TG mobilization and VLDL secretion, as well as promoting lipolysis in adipose tissue in the context of dexamethasone (DEX) injection, suggesting a tightly metabolic interaction between Angptl8 and glucocorticoid signaling.

## Results

### Hepatic *Angptl8* expression oscillates in a peripheral clock-independent manner

**Figure 1:**
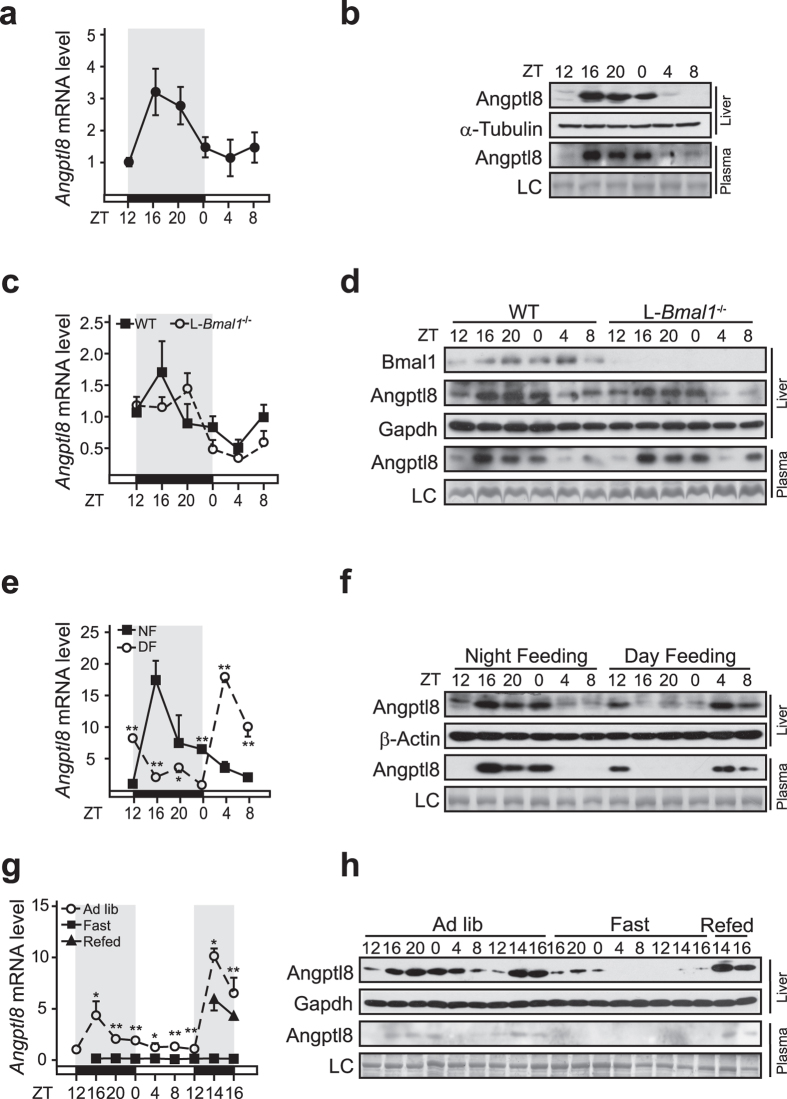
Food availability is a synchronizer of hepatic *Angptl8* oscillation. Ad libitum-fed liver-specific *Bmal1*-null mice and wild type littermates were euthanized at 4-hour intervals around the clock from Day1-ZT12 to Day2-ZT8. Liver and plasma samples were harvested. *Angptl8* mRNA and protein levels were measured by qPCR and immunoblot assay, respectively. Temporal profiles of hepatic *Angptl8* mRNA expression (**a**), as well as hepatic and plasma Angptl8 protein accumulation, LC: loading control (**b**) in wild-type mice. (**c**) Temporal expression of hepatic *Angptl8* in mice of indicated genotypes. (**d**) Hepatic and plasma Angptl8 protein accumulation profiles throughout the day. (**e,f**) Twelve-week-old male C57BL/6 mice were randomly separated into two groups: mice were provided food restrictedly at nighttime (ZT12-ZT0, NF); or at daytime (ZT0-ZT12, DF), and then animals were euthanized at 4-hour intervals from Day2-ZT12 to Day3-ZT8. Liver and plasma samples were harvested. Temporal expression of *Angptl8* was analyzed by qPCR (**e**) and immunoblot assay (**f)**. (**g,h**) Mice were randomly separated into three groups: for the fast group, mice were fasted from Day1-ZT12 to Day2-ZT20; for the refed group, mice were fasted for 24 h from Day1-ZT12 to Day2-ZT12 and then refed at Day2-ZT12; Mice fed ad libitum were served as controls. Animals were euthanized at 4-hour intervals around the clock from Day1-ZT12 to Day2-ZT20 as indicated. Temporal expression of *Angptl8* was analyzed by qPCR (**g**) or immunoblot assay (**h**). Data are represented as mean ± s.e.m, n = 3, *p < 0.05, **p < 0.01.

As plasma TG levels oscillate rhythmically in a 24-hour cycle^18^, and Angptl8 plays an important role in maintaining lipid metabolism^11^,^13^,^19^, we speculated that *Angptl8* expression may also exhibit circadian pattern. Thus, we checked the temporal expression of *Angptl8* in livers from mice euthanized at every 4-hour intervals throughout the day. Indeed, hepatic expression of *Angptl8* gene oscillated rhythmically with plateau near ZT16 and nadir around ZT4 (Fig. 1a,b). Considering that Bmal1, which serves as a core component of the circadian clock to govern the rhythmic expression of many genes^20^, is involved in regulating lipid homeostasis^21^, we wondered whether the circadian expression of *Angptl8* is controlled by Bmal1. To answer this question, we then examined the expression pattern of hepatic *Angptl8* in liver-specific *Bmal1-*knockout (L-*Baml1*^−/−^) mice. Unexpectedly, the rhythmic expression of *Angptl8* was almost unaffected by *Bmal1* deficiency (Fig. 1c). Consistently, Angptl8 protein profiles in the liver and plasma were nearly identical between L-*Baml1*^−/−^ mice and its wild-type littermates (Fig. 1d). Together, these results suggest that the expression of *Angptl8* oscillates diurnally in the liver, which is not controlled by hepatic core clock.

### Fasting and feeding signals modulate the expression of *Angptl8*

Since the expression of *Angptl8* is tightly associated with nutritional status^12^,^14^,^15^, we then performed food entrainment experiments to explore whether food availability is a potential synchronizer for *Angptl8* oscillation. As expected, daytime-restricted feeding (DF) completely reversed the phase of mRNA and protein accumulations of *Angptl8* in the liver, as well as its secretion pattern, compared to those with nighttime-feeding (NF, Fig. 1e,f). We further investigated the involvement of feeding style in synchronizing *Angptl8* expression by euthanizing mice at 4-hour intervals around the clock during 28-hour fasting initiated from Day1-ZT12 to Day2-ZT16 and 2-hour intervals after refeeding from Day2-ZT12 to Day2-ZT16. Compared to those in ad libitum-fed mice, the diurnal oscillations of *Angptl8* mRNA and Angptl8 protein accumulations were strikingly abolished by fasting, whereas were recovered by refeeding (Fig. 1g,h). Taken together, these data favor the conclusion that fasting and feeding signals play critical roles in generating the circadian oscillation of *Angptl8* expression.

### LXRα upregulates hepatic *Angptl8* expression in refed mice

**Figure 2:**
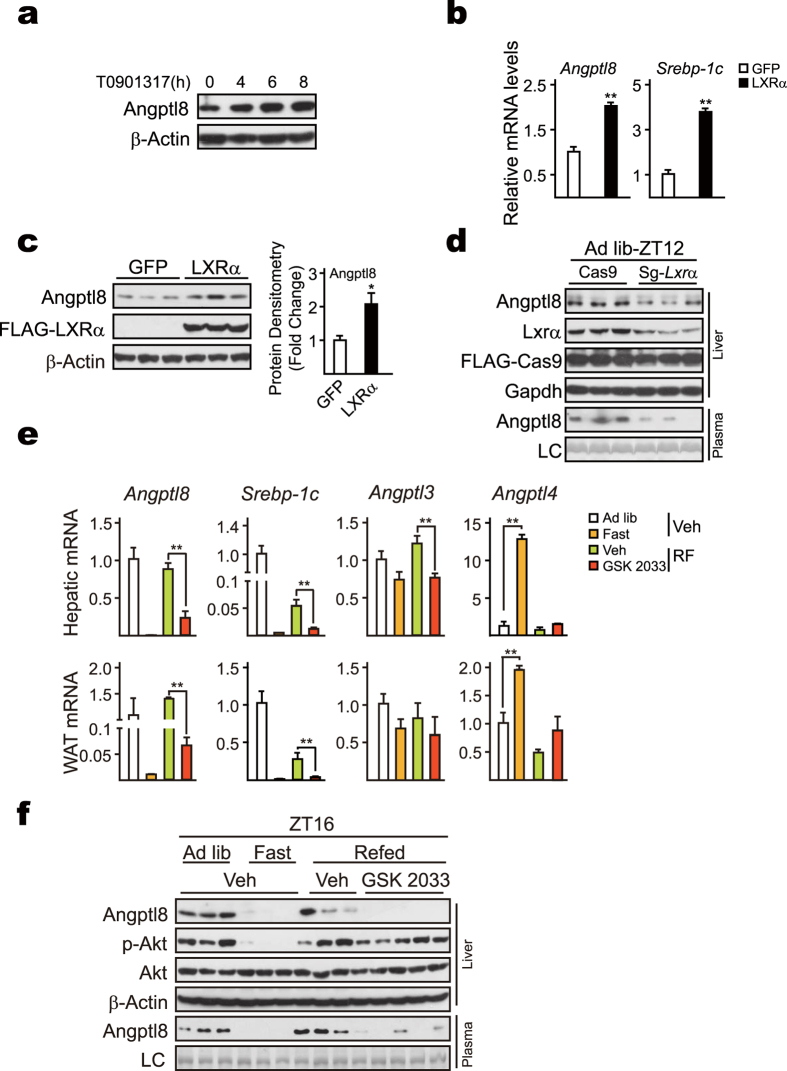
LXRα modulates *Angptl8* gene induction during refeeding. (**a**) T0901317 increases Angptl8 accumulations in a time-dependent manner. Mouse primary hepatocytes were treated with T0901317 (10 nM) for the indicated time points, after which immunoblot assay was performed. (**b,c**) Cultured primary hepatocytes were infected with Ad-GFP or Ad- LXRα. The infected cells were cultured O/N in serum-free M199 medium and then treated with T0901317 (10 nM) for 4 hours. *Angptl8* expression was analyzed by qPCR (**b**), and immunoblot assay (**c**), respectively. (**d**) Knockout of *Lxr*α decreases Angptl8 amounts *in vivo*. (**e,f**) Mice were randomly separated into four groups as indicated. For group 1, mice were fed ad libitum; for group 2, mice were fasted from Day1-ZT12 to Day2-ZT16; for two refed groups, mice were fasted from Day1-ZT12 to Day2-ZT12, followed by refed 4 h from Day2-ZT12 to Day2-ZT16. For the refed group, animals were intraperitoneally injected with vehicle (Veh) and GSK 2033 (20 mg/Kg) at Day2-ZT12, respectively. All animals were euthanized at Day2-ZT16. Liver and plasma samples were harvested. Temporal expression of *Angptl8* was analyzed by qPCR (**e**), and immunoblot assay (**f**), respectively. Data are represented as mean ± s.e.m, n = 3–5, *p < 0.05, **p < 0.01.

*Angptl8*, also named as RIFL (refeeding induced fat and liver)^15^, has been reported to be an insulin target gene and upregulated in HepG2 cells by the treatment of T0901317^17^, a ligand of liver X receptor α (LXRα) that regulates lipid metabolic gene expression in response to insulin. Indeed, we found that T0901317 promoted Angptl8 accumulation in a time-dependent manner in cultured primary hepatocytes (Fig. 2a). In addition, both *Angptl8* mRNA levels and Angptl8 protein levels were increased by overexpressing LXRα in primary hepatocytes (Fig. 2b,c). By contrast, CRISPR/Cas9-mediated knockout of *Lxrα* significantly attenuated Angptl8 protein amounts in mouse liver (Fig. 2d). Additionally, we applied GSK2033 to block the activation of LXRα to pursue the role of LXRα in regulating *Angptl8* expression during refeeding status. As expected, refeeding-induced upregulation of *Angptl8* mRNA accumulation was significantly attenuated in the liver and WAT by GSK2033 administration (Fig. 2e). Correspondingly, both hepatic and plasma Angptl8 protein levels were dramatically decreased (Fig. 2f). Together, these results demonstrate that LXRα plays a critical role in regulating *Angptl8* expression during feeding periods.

### Identification of LXREs within the promoter region of *Angptl8* gene

**Figure 3:**
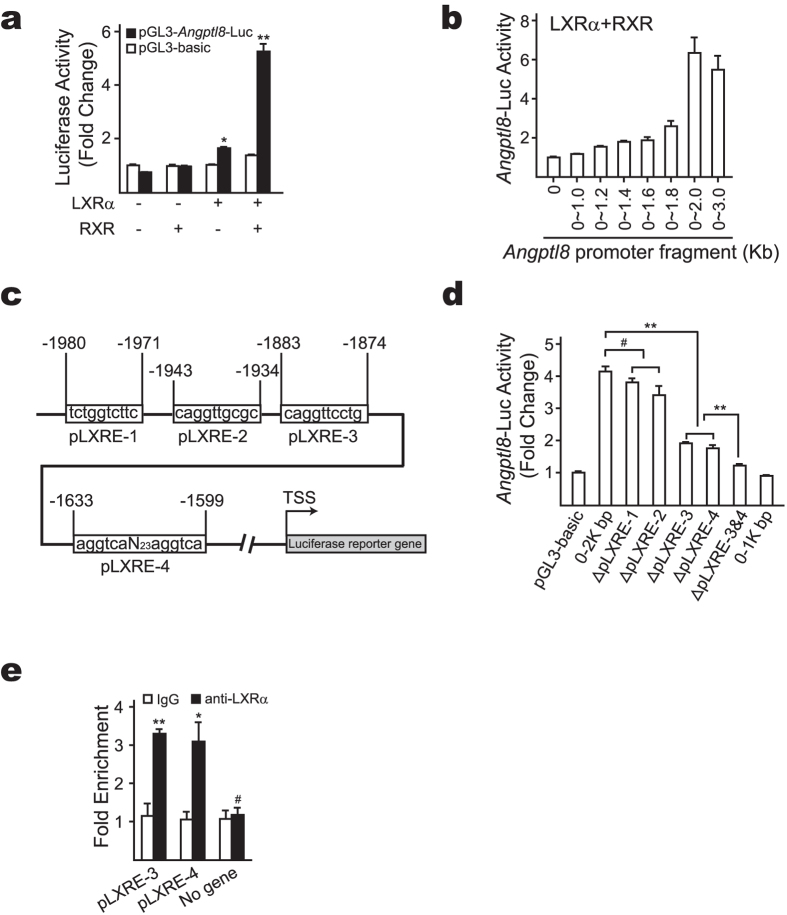
Identification of two non-canonical LXR response elements. (**a**) LXRα and RXR increase *Angptl8*-Luc activity. Indicated plasmids were co-transfected into HEK293T cells as specified and luciferase activity was measured and calculated as described in Methods. (**b**) *Angptl8* promoter truncation analysis. Reporter plasmids driven by different *Angptl8-*promoter fragments, LXRα, RXR and β-gal were co-transfected into HEK293T cells as indicated, then cells were lysed and assayed for firefly luciferase and β-gal activities. (**c**) Graphic of putative LXREs (pLXREs) in the promoter region of *Angptl8.* (**d**) *Angptl8* promoter deletion analysis. Reporter plasmids driven by different *Angptl8*-promoter regions (Δ: deletion), as well as LXRα, RXR and β-gal were co-transfected into HEK293T cells as indicated, then cells were lysed and assayed for firefly luciferase and β-gal activities. (**e**) ChIP analysis of the occupancy of LXRα on *Angptl8* pLXRE sites in livers of mice euthanized at ZT16. No gene serves as negative control. Data are represented as mean ± s.e.m, n = 3, *p < 0.05, **p < 0.01, # no significant difference.

To investigate the underlying mechanism of LXRα regulation of Angptl8 expression, we constructed an *Angptl8* promoter-driven luciferase reporter plasmid. As luciferase reporter assay results suggested, overexpression of LXRα together with retinoid X receptor (RXR) enhanced the activity of *Angptl8* promoter-driven luciferase reporter (Fig. 3a). Since promoter analysis failed to identify any canonical LXR response elements (LXREs) in this promoter region of *Angptl8*, we then performed promoter-truncation analysis to reveal that the region from −1.8 to −2 kbp of *Angptl8* promoter was mainly responsible for LXRα-induced *Angptl8*-Luc expression (Fig. 3b). To further identify the binding site(s) of LXRα, we then made a series of deletions around four putative LXREs (denoted as pLXRE-1 to pLXRE-4, Fig. 3c). The results from luciferase reporter assay indicated that pLXRE-3 and pLXRE-4 were required for LXRα induction of *Angptl8*-Luc activity (Fig. 3d). Supporting these results, ChIP assay demonstrated that LXRα was present on these sites at ZT16 when *Angptl8* mRNA level was near its plateau (Fig. 3e).

### Glucocorticoids negatively regulate *Angptl8* expression during fasting

**Figure 4:**
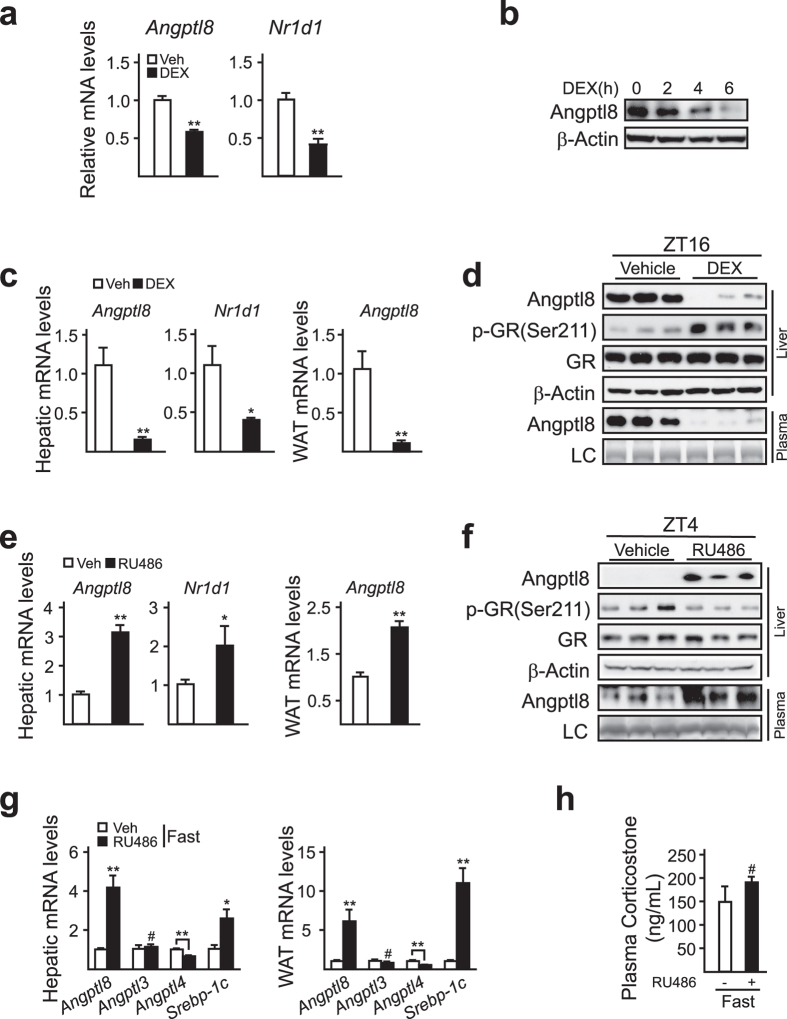
Glucocorticoids suppress *Angptl8* expression during fasting. (**a**) Quantitative PCR analysis of *Angptl8* mRNA levels in dexamethasone (DEX, 10 nM, 4 h) treated primary hepatocytes. (**b**) Immunoblotting analysis of Angptl8 protein levels in primary hepatocytes treated with DEX (10 nM) for the indicated time points. (**c,d**) Ad libitum-fed C57BL/6 mice were intraperitoneally injected with vehicle (PBS) or dexamethasone (DEX, 2 mg/Kg) at ZT12, and then animals were euthanized at ZT16. Temporal expression of *Angptl8* was analyzed by qPCR (**c**), or immunoblot assay (**d**). (**e,f**) Ad libitum-fed mice were intraperitoneal injected with vehicle (PBS) or RU486 (20 mg/Kg, n = 5) at ZT0, and then animals were euthanized at ZT4. Temporal expression of *Angptl8* was analyzed by qPCR (**e**), or immunoblot assay (**f)**. (**g,h**) Mice were intraperitoneally injected with vehicle (PBS) or RU486 (20 mg/Kg) at ZT12, then animals were kept fasting and euthanized at ZT16. qPCR analysis of *Angptl3, 4, 8* and *Srebp-1c* mRNA levels (**g**). Plasma glucocorticoid levels (**h**). Data are represented as mean ± s.e.m, * *p* < 0.05, ** *p* < 0.01, # no significant difference.

To explore the mechanism of fasting-induced *Angptl8* repression, we analyzed the promoter region of *Angptl8* and noticed a palindromic sequence (cctcNNggag) of negative glucocorticoid response element (nGRE) that locates between −1096 bp and −1105 bp upstream of the transcriptional start site (TSS) of *Angptl8* gene. Coincidently, plasma glucocorticoid profile was opposite with the expression pattern of *Angptl8* gene *in vivo* (Fig. 1a)^22^. We thus examined the role of glucocorticoids in regulating *Angptl8* expression. As shown in Fig. 4a, dexamethasone (DEX) treatment repressed the mRNA levels of *Angptl8* and *Nr1d1*, a known glucocorticoid-inhibited gene^23^, in primary mouse hepatocytes. Correspondingly, Angptl8 protein amounts decreased in a time-dependent manner after DEX treatment (Fig. 4b). In line with these *in vitro* results, *Angptl8* expression was suppressed in the liver and white adipose tissues (WAT) from mice intraperitoneally injected with DEX at ZT12, when plasma levels of glucocorticoids begin to decrease^22^, and euthanized around ZT16, compared to those injected with control vehicle (Fig. 4c,d). To further verify the role of glucocorticoids in suppressing *Angptl8* expression, we applied mifepristone (RU486), a synthetic anti-glucocorticoid drug, to block the glucocorticoid signals *in vivo*. As expected, RU486 administration significantly attenuates fasting-induced *Angptl8* suppression both in the liver and WAT (Fig. 4e–g), without influencing plasma glucocorticoid levels (Fig. 4h). Together, these results suggest that glucocorticoid signals mediate fasting-induced *Angptl8* suppression.

### GR mediates the inhibitory effect of glucocorticoids on *Angptl8* expression

**Figure 5:**
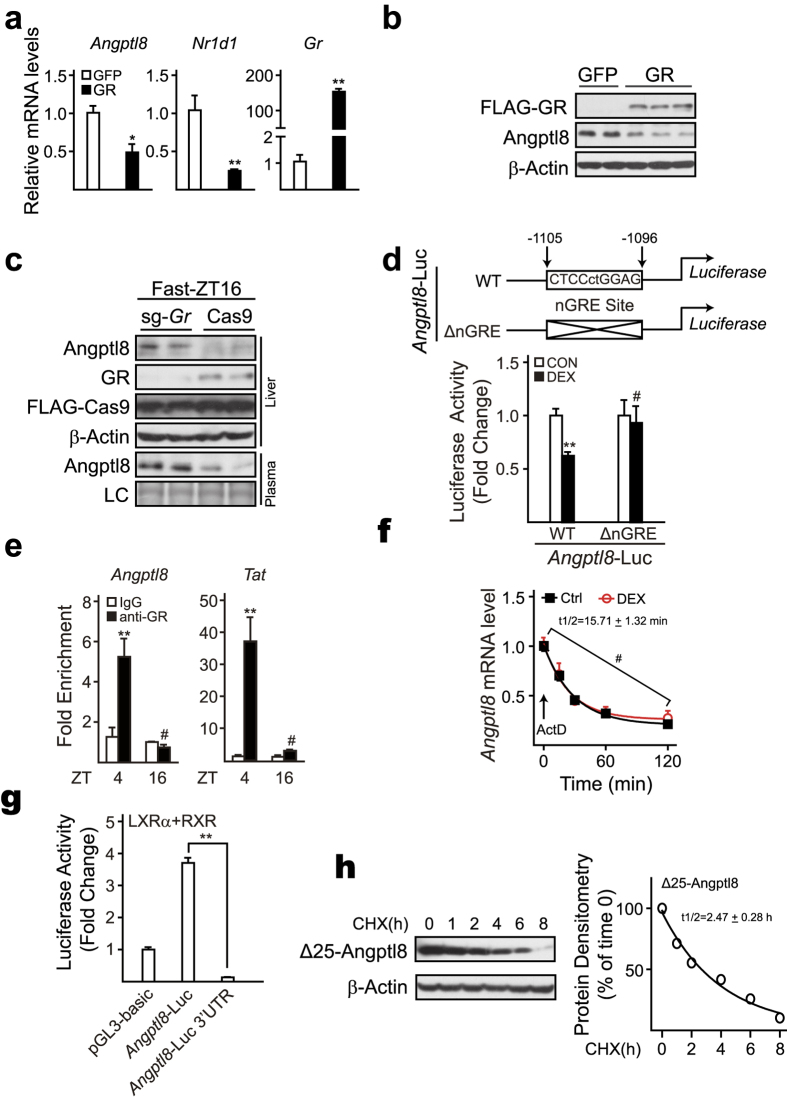
Identification of nGRE element in *Angptl8* promoter region. Cultured mouse primary hepatocytes were infected with Ad-GFP and Ad- GR virus, respectively. The infected cells were cultured overnight (O/N) in serum-free M199 and then treated with DEX (10 nM) for 4 hours. *Angptl8* mRNA levels (**a**), and Angptl8 protein amounts (**b**) were analyzed by qPCR and immunoblot, respectively. (**c**) Knockout of *Gr* ameliorates Angptl8 reduction caused by fasting. Cas9 alone or sg-*Gr*-Cas9 adenoviruses were delivered into livers of adult C57BL/6 mice by tail-vein injection on Day1, and then animals were fasted at Day15-ZT12, euthanized at Day15-ZT16. Liver and plasma samples were harvested and immunoblot assay was performed to detect Angptl8 protein levels. (**d**) The nGRE site is necessary for *Angptl8* to response to DEX treatment. Reporter plasmid (*Angptl8*-WT-Luc or *Angptl8*-ΔnGRE-Luc, up) and RSV-β-gal plasmid were co-transfected into HEK293T cells. The transfected cells were cultured O/N in serum-free DMEM and then treated with vehicle (PBS) or DEX (10 nM) for 4 hours. Luciferase activities were measured and normalized to β-gal activity (bottom). (**e**) ChIP analysis of the occupancy of GR on *Angptl8* nGRE site in livers of ad libitum-fed mice euthanized at ZT4 and ZT16. *Tat* here serves as a positive control. (**f**) Half-life of *Angptl8* mRNA. Cultured primary hepatocytes were treated with actinomycin D (ActD, 5 ug/uL) or ActD plus DEX (10 nM) for the indicated time points. *Angptl8* mRNA levels were then analyzed by qPCR and *Angptl8* mRNA half-life was calculated by using one phase decay equation. (**g**) 3′-UTR of *Angptl8* takes responsibility for *Angptl8* mRNA instability. HEK293T cells were transfected with *Angptl8*-Luc and *Angptl8-*Luc*-*3′UTR, respectively. The transfected cells were left overnight, and then lysed and assayed for firefly luciferase and β-gal activities. (**h**) HEK293T cells were transfected with Δ25- Angptl8 (Angptl8 without signal peptide) plasmids, then cultured O/N in serum-free DMEM and treated with cycloheximide (CHX, 10 ug/mL) for the indicated time points, after which immunoblot assay was performed (left) and Δ25- Angptl8 half-life was calculated by using one phase decay equation (right). Data are represented as mean ± s.e.m, n = 3, *p < 0.05, **p < 0.01, # no significant difference.

Since the biological effects of glucocorticoids are mainly conferred by their cognate intracellular receptor, glucocorticoid receptor (GR), we then examined the necessity of GR in the suppression of *Angptl8* expression induced by glucocorticoids. Indeed, adenoviral overexpression of GR markedly augmented the reduction of *Angptl8* expression induced by DEX-treatment in cultured primary hepatocytes (Fig. 5a,b). We further verified such effect *in vivo* by using adenovirus-delivered CRISPR/Cas9-GR system in mouse liver. As expected, Ad-CRISPR/Cas9-mediated liver-specific *Gr* deficiency significantly diminished fasting-induced suppression of hepatic *Angptl8* expression (Fig. 5c). To map the binding site(s) of GR on *Angptl8* promoter, we then constructed a plasmid expressing a luciferase reporter driven by either the wide-type promoter region of *Angptl8* gene (2000 bp upstream ahead of the TSS, *Angptl8-*WT-Luc) or a corresponding nGRE-deletion mutant one (*Angptl8*-ΔnGRE-Luc, Fig. 5d, up). As a result, DEX significantly reduced the activity of *Angptl8*-WT-Luc in HEK293T cells, but not that of *Angptl8*-ΔnGRE-Luc (Fig. 5d, bottom). Consistently, results of chromatin immunoprecipitation (ChIP) assay demonstrated that GR was dynamically recruited to the nGRE site within *Angptl8* promoter region in the liver (Fig. 5e), which accounts for the different transcriptional level of *Angptl8* between ZT4 and ZT16. Conclusively, these results confirm that GR binds directly to the nGRE element within *Angptl8* promoter region to mediate the inhibitory effect of glucocorticoids on *Angptl8* expression.

### *Angptl8* mRNA decays rapidly after transcription

Considering that mRNA/protein instability is the nature of almost all of the circadian genes, the rapidly turnover of *Angptl8* expression between fasting/feeding transitions promoted us to examine the half-life of its mRNA. Supporting this notion, *Angptl8* mRNA levels rapidly dropped after actinomycin D (ActD) treatment and its half-life was just approximately 15.71 min, which was independent of glucocorticoid signaling (Fig. 5f). As the 3′- untranslated region (3′-UTR) has been shown to play a major role in harboring determinants to control mRNA decay^24^,^25^, we constructed a chimeric plasmid that replaces the luciferase reporter 3′-UTR with that of mouse *Angptl8* (pGL3-*Angptl8*–3′-UTR) to determine whether the *Angptl8* 3′-UTR is involved in the regulation of its mRNA stability. Strikingly, the luciferase activity of pGL3-*Angptl8*–3′-UTR was almost abolished, compared to that of control luciferase reporter (Fig. 5g). In addition, we examined Angptl8 protein stability with a plasmid encoding Angptl8 protein without signal peptide (Δ25- Angptl8) to avoid the influence of secretion. As shown in Fig. 5h, Angptl8 protein was relatively stable, with the half-life of approximately 2.47 h, compared to its mRNA stability. Together, these results suggest that the *Angptl8* mRNA instability contributes to the rapid response of *Angptl8* expression to fasting/feeding transitions, while the Angptl8 protein stability makes it possess the capability to exert its function after secretion.

### Effects of hepatic overexpression of Angptl8 on glucocorticoid-regulated lipid metabolism

**Figure 6:**
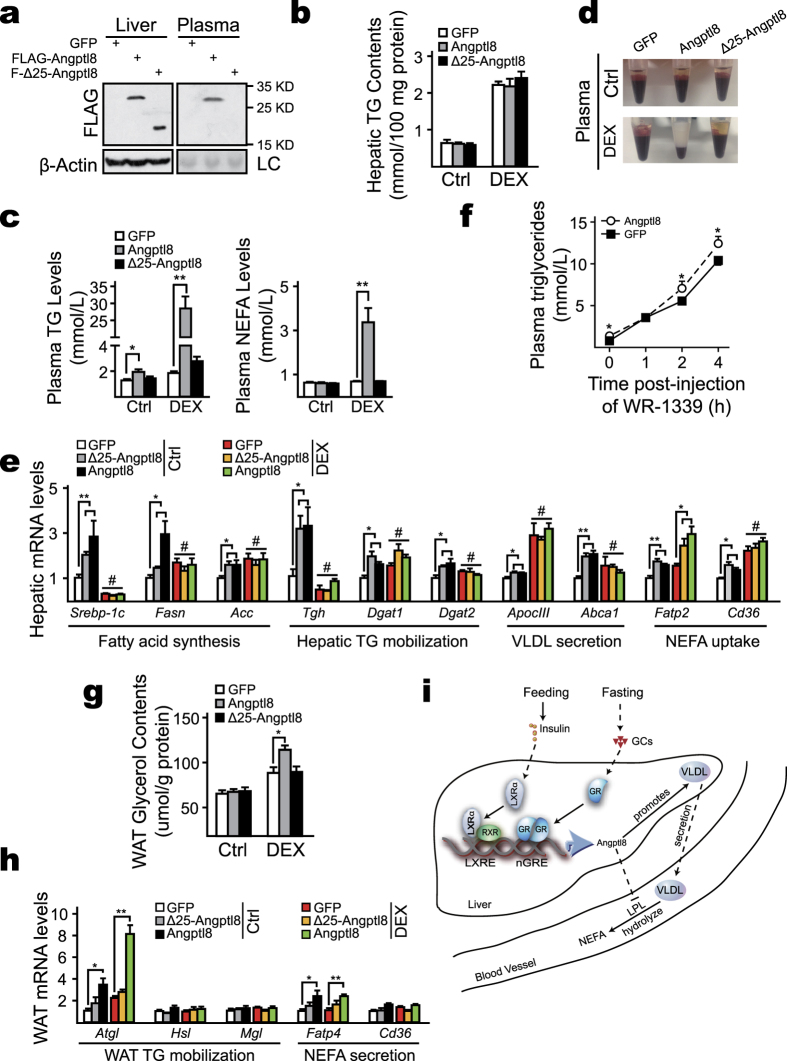
Metabolic interaction between Angptl8 and glucocorticoid signaling. Ad-GFP, Ad- Angptl8 or Ad-Δ25- Angptl8 adenoviruses were delivered into mice via tail-vein injection on Day1. Mice were treated with vehicle (PBS) or DEX (100 mg/Kg) by intraperitoneal injection once-daily for 3 days from Day4 to Day6, and then animals were euthanized on Day10. (**a**) Immunoblotting analysis of hepatic and plasma Angptl8 and Δ25- Angptl8. (**b**) Measurement of hepatic TG contents. (**c**) Measurement of plasma TG and NEFA levels of indicated virus-infected mice. (**d**) A picture of plasma shows that ectopic expression of Angptl8 results in cream-like plasma in DEX-treated mice. (**e**) Quantitative PCR analysis of mRNA levels of hepatic genes involved in lipid metabolism. (**f**) Plasma TG levels post-injection of Triton WR-1339 (500 mg/Kg) at the indicated time points. (**g**) Overexpressing Angptl8 promotes lipolysis in adipocytes in DEX-treated mice n = 4–8, *p < 0.05, **p < 0.01, # no significant difference. (**h**) Overexpressing Angptl8 affects gene expression involved in lipolysis in WAT. (**i**) Hypothetical model showing the mechanism of fasting and feeding signals modulate *Angptl8* expression in mouse liver. Postprandially, the liver X receptor alpha (LXRα) heterodimerizes with retinoid X receptor (RXR) and binds to the liver X receptor response element (LXRE) in the promoter of *Angptl8* to initiate its transcription. During fasting state, elevated glucocorticoids suppress *Angptl8* transcription by activating glucocorticoid receptor (GR) and subsequent binding of GR dimmers to the negative glucocorticoid response element (nGRE) in the promoter of *Angptl8*. Elevated Angptl8 increases plasma TG concentration, whereby promoting hepatic VLDL secretion and decreasing VLDL hydrolization via inhibiting LPL activity.

Considering that chronically elevated glucocorticoid levels lead to non-alcoholic fatty liver^26^,^27^, whereas the expression of *Angptl8* is inhibited by glucocorticoid signal, we wondered whether Angptl8 plays a role in this regard. To answer this question, we overexpressed GFP, Angptl8 or Δ25- Angptl8 in the liver by tail-vein delivery of corresponding adenoviruses on day 1, and then injected these mice with either DEX or PBS once-daily for three days from day 4 to day 6. Expression of Angptl8 and Δ25- Angptl8 were readily detectable after 10-days post-injection (Fig. 6a). Surprisingly, overexpression of Angptl8 didn’t ameliorate fatty liver development in the mice with DEX injection (Fig. 6b), whereas, plasma TG and NEFA levels were dramatically elevated in these animals (Fig. 6c), resulting in cream-like plasma (Fig. 6d). These data imply a key role of Angptl8 in plasma TG clearance and adipose TG mobilization in the context of DEX injection. Supporting this idea, we found that many genes involved in fatty acid synthesis, NEFA uptake, VLDL secretion were upregulated in the liver by overexpressing Angptl8 (Fig. 6e), which accounts for the accelerated hepatic VLDL secretion rate (Fig. 6f). Intriguingly, ectopic expression of Δ25-Angptl8 exhibited similar effect as Angptl8 overexpression (Fig. 6e), which implies an intracellular functional role of Angptl8 in modulating gene transcription. Moreover, overexpression of Angptl8 exerted no significant influence on transcription of gluconeogenesis-related genes, e.g. *G6p* and *Pepck* (data not shown), which is consistent with the knowledge that Angptl8 does not impair glucose homeostasis^13^,^19^. Of note, overexpression of Angptl8 promotes lipolysis in adipocytes with DEX-treatment (Fig. 6g). Supporting these results, we noticed that *Atgl* and *Fatp4*, two genes involved in adipose TG mobilization and NEFA secretion, respectively, were upregulated in these regards (Fig. 6h). Taken together, these results confer a vital role of Angptl8 in modulating TG homeostasis and suggest a tightly metabolic interaction between Angptl8 and glucocorticoid signaling.

## Discussion

Angptl8 accumulation is intimately linked with circulating TG contents, and emerging evidence implies a key role of Angptl8 in the metabolic transition between fasting and feeding^13^. In the present study, we show that LXRα heterodimerizes with RXR to initiate the transcription of *Angptl8* induced by refeeding. On the other hand, glucocorticoids suppress *Angptl8* transcription by activating and subsequent recruiting GR dimmers to the nGRE element within *Angptl8* promoter region during fasting. In addition, our data confer the specific role of Angptl8 in mediating plasma TG clearance, as well as modulating VLDL secretion (Fig. 6i).

The instability of *Angptl8* mRNA (~15.71 min, Fig. 5f) is vital for *Angptl8* oscillations and functions. Specifically, it allows the rapid response of *Angptl8* expression to fasting-feeding transition, which is involved in the generation of the circadian rhythm of its mRNA, as well as intracellular and plasma protein oscillations. Moreover, this nature of *Angptl8* mRNA fits its role in regulating lipid metabolism that is quickly and dramatically altered by eating. On the other hand, the relative high stability (~2.47 h, Fig. 5h) permits Angptl8 protein to be secreted and exert its function.

Although the circadian oscillation of *Angptl8* expression is not controlled directly by the peripheral core clock machinery, it is generated by feeding behaviors that are governed by the central clock locating in the suprachiasmatic nucleus^28^. Furthermore, the production and secretion of glucocorticoids, which is the key inhibitory regulator of *Angptl8* expression, are under the control of the circadian clock^29^. Thus, the rhythmic expression of *Angptl8* is affected indirectly by the molecular clock. In addition, we notice that hepatic Angptl8 protein abundances are slightly reduced in liver-specific *Bmal*1-knockout mice, compared to those in WT littermates (Fig. 1d), which may be due to the reduced protein synthesis caused by Bmal1 deficiency^30^.

LXRs (α/β) have been shown to play critical roles in the transcriptional regulation of lipid metabolism. For instance, activation of LXRs increases hepatic fatty acid synthesis, VLDL secretion to result in hypertriglyceridemia^31^. Intriguingly, both *Angptl8* and *Angptl3* are primary targets of LXRs^32^, and Angptl3 function together with Angptl8 to inhibit plasma TG clearance^11^. On the other hand, *Angptl3* expression is modestly altered by feeding behaviors^33^, whereas *Angptl8* is dramatically affected by nutritional status^15^. It seems that Angptl8 functions as a responser to fasting and feeding signals and cooperates with Angptl3 to maintain lipid homeostasis. Taken together, these evidences reveal an intuitively mechanism of Angptl3/ Angptl8-mediated hypertriglyceridemia induced by LXRs.

An intriguing cognition seems clear about how glucocorticoids and LXRs regulate plasma TG trafficking whereby modulating the expression of *Angptl3*, *4*, and *8*. Although no significant change happens on *Angptl3* transcription during fasting (Fig. 2e), *Angptl8* is dramatically suppressed Fig. 1g–h and Fig. 2e, whereas *Angptl4* is upregulated due to the elevated circulating glucocorticoid levels^34^. On one hand, reduced amounts of the Angptl8 protein augments LPL activity specifically in heart and skeletal muscles^35^, and thus promotes the hydrolysis of VLDL-TG to result in the increased circulating NEFA concentrations. On the other hand, increased amounts of Angptl4 protein decreases LPL activity in an adipose-specific manner^36^, and promotes intracellular lipolysis in adipocytes^37^. Together, decreased adipose-LPL activity and enhanced muscle-LPL activity direct the flux of circulating TG toward peripheral tissues for utilization. Such fasting scenario is completed reversed under feeding status. Briefly, feeding-induced activation of LXRs and decrease of circulating glucocorticoids lead to the upregulation of *Angptl8* and downregulation of *Angptl4*. As a result, plasma TG is directed to adipose tissues for storage. Supporting this notion, Angptl3 has been suggested to be involved in the redirection of TG to adipose tissue for storage during feeding, whereas the postprandial increase in TG delivery to adipose tissue is abolished in mice lacking *Angptl8*^7^.

Consistent with previous report that the absence of *Angptl8* profoundly disrupts VLDL secretion^13^, our data demonstrate that overexpression of Angptl8 in mouse livers upregulates hepatic genes involved in lipid metabolism (Fig. 6e), which considerably accelerates VLDL secretion (Fig. 6f). However, such effect of Angptl8 overexpression is almost abolished by DEX injection (Fig. 6e), which may be due to the dominant impact of DEX on these gene expressions diminishes the difference between Ad-GFP- and Ad- Angptl8-infected mice. Although the secretion rate was accelerated, hepatic TG contents were identical between GFP and Angptl8 overexpressed mice. One of the possible explanations is that the whole process of lipid metabolism from NEFA uptake to hepatic TG synthesis, VLDL-TG incorporation and secretion is accelerated by Angptl8 overexpression, which results in the balance of TG homeostasis in the liver, but the great TG accumulation in the plasma. In addition, we notice that *Angptl8* deficiency doesn’t impair the expression of lipid biosynthesis genes, such as *Srebp-1c* and *Acc*^13^, which is inconsistent with the results of overexpressing Angptl8 described in this study. One possible explanation for this discrepancy is that there may be other factors that can compensate the intracellular function of Angptl8.

Of note, ectopic expression of wild-type Angptl8, rather than a mutant one lacking the signal peptide responsible for secretion (Δ25*-*Angptl8), results in markedly increased plasma TG and NEFA levels in DEX-treated mice (Fig. 6c,d). One of the most likely explanations for this strong phenotype is that the highly expressed Angptl8 and Angptl4 in such circumstance diminish plasma TG clearance to cause sustained circulating TG accumulation, as suggested by the Angptl3-4-8 model^38^. Besides, overexpression of Angptl8 upregulates *Atgl* and *Fatp4* gene expression (Fig. 6h) to prompt lipolysis in adipocytes in DEX-injected mice (Fig. 6g) to result in the dramatically elevated plasma NEFA levels (Fig. 6c). These data further suggest a close metabolic interaction between Angptl8 and glucocorticoid signaling in adipose tissue. Moreover, ectopically overexpressing Δ25*-*Angptl8 exhibits similar effect on lipid metabolism-related gene expression as Angptl8 (Fig. 6e), implying an intracellular functional role of Angptl8 in modulating gene transcription. Further investigations are needed to explore the functional role of Angptl8 in these regards.

## Materials and Methods

### Mice and treatments

Eight- to twelve-week-old C57BL/6 J male mice were purchased from Shanghai Laboratory Animal Centre (China) and housed in the animal facility at Shanghai Institutes for Biological Sciences (SIBS). Mice were maintained on a 12-h light/12-h dark cycle for at least 2 weeks before study and had free access to water and regular diet (12% fat/68% carbohydrate/20% protein). For time-restricted feeding experiment: mice under daytime-restricted feeding (DF) were provided food during the entire light period (7:00 AM–7:00 PM), whereas nighttime-restricted feeding (NF) mice were provided food only at dark time (7:00 PM–7:00 AM). Experimental schedule of fasting and refeeding experiments has been described^22^. For hepatic Angptl8 overexpression study, recombinant adenoviruses were delivered into mice by tail vein injection, and then animals were intraperitoneally injected with PBS or DEX (100 mg/Kg) for successive three days from day 4 to day 6 and euthanized on day 10 post-injection. For other *in vivo* experiments, mice were treated as described specifically in Figure Legends. All research procedures were performed in accordance with the guidelines and regulations, as well as ethically approved by the Animal Care and Use Committee of the Institute for Nutritional Sciences.

### Cell culture

HEK293T cells were purchased from American Type Culture Collection (ATCC). Mouse primary hepatocytes were isolated by using collagenase (Sigma) and plated on dishes pre-coated with rat tail tendon collagen (Sigma) according to manufacturers’ instructions. HEK293T cells and primary hepatocytes were cultured in Dulbecco’s Modified Eagle’s Medium (DMEM) and medium 199 (M199), respectively. Complete medium containing 10% fetal bovine serum and 1% penicillin/streptomycin. HEK293T cells were transfected by using PEI (Sigma) reagent according to manufacturers’ instructions. Briefly, plasmid (ug) and PEI reagent (1 ug/uL) were mixed in a ratio of 1:3, and then incubated for 15 min at room temperature. Medium was changed into serum-free DMEM, and then PEI/Plasmid transfection complexes were added into wells. Cells were lysed for western blot or luciferase activity analysis after 24 hours post-transfection. Primary hepatocytes were synchronized by serum starvation for overnight and then infected by adenovirus as specified.

### Plasmid

The expression plasmids used in this study were generated according to standard molecular biology techniques. For luciferase reporter plasmid construction, the mouse *Angptl8* genomic fragments were amplified by polymerase chain reaction (PCR) with specific primers. The amplified PCR products were separated using DNA agarose gel and recovered using QIAquick Gel Extraction Kit (cat. 28706), then digested with corresponding restriction enzymes. The prepared PCR products were then linked into pGL3-basic reporter plasmid, which was previously digested with the same restriction enzymes as used to digest PCR products. *Angptl8*, *Lxrα*, *et al.* were amplified and cloned into pcDNA plasmid by using the same method as mentioned above. All the plasmids were subjected to sequencing for verification. Primers used for cloning were provided in Supplementary Table 1.

### Immunoblot Assay

For immunoblot analysis, cultured cells or harvested liver tissues were lysed in RIPA buffer containing proteinase inhibitors (PMSF, Cocktail and Phosphatase Inhibitors), followed by centrifuging at 12,000 r.p.m for 15 min at 4 °C. Supernatants were collected and protein concentrations were determined by using BCA protein assay kit (Pierce, 23225) according to manufacturers’ instruction. Took same amounts of total protein from the left supernatants, then added corresponding volume of lysis buffer and 5x SDS loading buffer to make the final protein concentration identical, then boiled for 10 min. Identical amounts of protein were subjected to SDS-PAGE electrophoresis and transferred to methanol-activated polyvinylidene fluoride membranes (PVDF, Millipore). Membranes were then blocked with 5% milk for 1 h at room temperature and incubated overnight with corresponding primary antibodies at 4 °C, then with secondary antibodies for 1 h at room temperature. Finally, the protein bands were developed by using ECL western blotting substrate (Thermo Pierce) and visualized with western film processor. Primary antibodies used in this study were as follows: Bmal1 (ab3350, Abcam, 1:1000), pSer473-Akt (#9271, CST, 1:1000), Akt (#9272, CST, 1:1000), GR (ab3578, Abcam, 1:1000), LXRα (ab41902, Abcam, 1; 1000), FLAG-HRP (A8592, Sigma, 1:5000), Angptl8 (generated by using the epitope of EFETLKARADKQ, 1:500), and β-Actin (#4967, CST, 1:6000).

### Adenoviruses

We applied CRISPR/Cas9 system to explore the effects of GR and LXRα on *Angptl8* expression. Single-guide RNAs used to mediate site-specific gene mutation by Cas9 were designed according to previously described^39^. We synthesized the designed sgRNAs and inserted them into the reconstructed pShuttle plasmids that contain U6 promoters for sgRNA expression and Cas9 coding sequence. Adenoviruses encoding Angptl8, GR, LXRα, green fluorescent protein (GFP), gene specific single-guide RNA (sg-GR, sg-LXRα) and Cas9 alone (sg-Cas9) were generated using AdEasy system as previously described^40^. Sequences of sg-RNAs used to mediate gene specific knock-out were as follows:

sgRNA-GR-Guide: CACCGACGGCTGGTCGACCTATTG,

sgRNA-GR-Comp: AAACCAATAGGTCGACCAGCCGTC;

sgRNA-LXRα-Guide: CACCGTGGGCCAAGGCGTGACGCGC

sgRNA-LXRα-Comp: AAACGCGCGTCACGCCTTGGCCCAC.

### Total RNA isolation and qPCR analysis

Total RNAs from liver or cultured primary hepatocytes were extracted using Trizol reagents (Invitrogen). The mRNAs were then reverse-transcripted into cDNA by the primer script^TM^ RT reagent kit with gDNA eraser (Takara) according to manufacturers’ protocol. Relative mRNA levels were determined by SYBR Green RT-PCR kit (Takara) with ABIPRISM 7900HT sequence detector (Perkin Elmer). Real-time PCR data were analyzed by the comparative C_T_ method^41^. Ribosomal L32 mRNA levels were used as internal control. Primers used for qPCR analysis were provided in Supplementary Table 2.

### Chromatin Immunoprecipitation (ChIP)

Liver tissues were cross-linked with 1% formaldehyde. GR or LXRα antibodies were used for immunoprecipitation (IP) along with rabbit IgG for negative controls. After removing cross-link, DNA was extracted using phenol–chloroform and ethanol precipitated. Target promoters were analyzed using SYBR Green Real-time PCR and normalized to input chromatin signals. Primer sequences used in these experiments were provided as follows (5′-3′):

GR -ChIP-F: TAAGGTGGTCCCACCAGTAG;

GR -ChIP-R: CAGGTTCCGGTTCTCTTTGT.

LXRα-ChIP-F3: GTGTGGGTACACGAAGAGCA;

LXRα-ChIP-R3: GAGGTTCCACCCACTGTCTG.

LXRα-ChIP-F4: TCCTGGTCTACATAGCAAGT;

LXRα-ChIP-R4: CTAAATAGCAAACTCAGATC.

ChIP primers used for *Tat* and *No Gene* were as previously described^42^,^43^.

### Luciferase activity assay

Cells were collected after 24 hours post-transfection and lysed using lysis buffer (Gly-gly buffer: 25 mM Gly-gly, pH 7.8; 15 mM MgSO4; 4 mM EGTA, pH 7.8; 1 mM DTT and 1% V/V Triton X-100). For 24-well plate, we added 150 uL of lysis buffer to each well, and then shook gently for 20 minutes by transference decoloring shaker. Mixed 50 uL of lysate with 50 uL of assay mix (20 mM Gly-gly, pH 7.8; 12 mM MgSO4; 3 mM EGTA, pH 7.8; 0.2 mM Potassium Phosphate, pH 7.8; 2 mM ATP; 1.5 mM DTT and 1.25 mg/mL firefly luciferin) in a well of a black 96-well plate. Fluorescence intensity was measured using the equipment of Luninoscan Ascent (Thermo). For β-gal assay to normalize the luciferase assay results, another 50 μl per well of lysate was taken to be mixed with 50 μl per well of β-gal solution, incubated at room temperature until color developed, and then read A420 on a plate reader (Epoch Microplate Spectrophotometer, BioTek).

### TG and NEFA measurement

To measure hepatic TG, we balanced approximately 100 mg of liver tissue and added 500 uL of cold PBS, then homogenized in a 1.5 mL eppendorf tube on ice. 200 uL of homogenates were transferred into a new tube and mixed with 800 uL of chloroform/methanol (2:1, v/v) adequately, then followed by centrifuging at 2500 r.p.m for 10 min at 4 °C. Quitted upper water-phase and transferred lower organic phase into a new tube, evaporated to dryness overnight in a chemical hood. The evaporated samples were then resuspended in 800 uL of EtOH-Triton X-100 (1% Triton X-100 in absolute ethanol), then TG contents were measured using commercial assay kit (SHENSUO UNF, China). Remainder of each homogenate was used to determine protein concentrations by using BCA protein assay kit (Pierce, 23225) according to manufacturers’ instruction. The final TG contents were normalized with corresponding protein concentrations. For plasma TG and NEFA measurement, bloods were harvested after mice were euthanized and plasma samples were prepared. Plasma TG and NEFA were determined with TG kit (SHENSUO UNF, China) and Wako HR Series NEFA-HR kit (Wako Pure Chemical Industries, Ltd), respectively.

### Statistical Analyses

Results were represented as mean ± s.e.m. The comparisons of two groups of mice or different primary hepatocytes preparations were carried out using two-tailed unpaired Student’s *t* test. Differences were considered statistically significant at *P* < 0.05. No statistical methods were used to predetermine sample size. All experiments were performed on at least two independent occasions.

## Additional Information

**How to cite this article**: Dang, F. *et al.* Fasting and Feeding Signals Control the Oscillatory Expression of *Angptl8* to Modulate Lipid Metabolism. *Sci. Rep.* **6**, 36926; doi: 10.1038/srep36926 (2016).

**Publisher’s note**: Springer Nature remains neutral with regard to jurisdictional claims in published maps and institutional affiliations.

## Supplementary Material

Supplementary Information
