# Supplementary material for: Fasting and Feeding Signals Control the Oscillatory Expression of Angptl8 to Modulate Lipid Metabolism
Source: Sci Rep. 2016 Nov 15;6:36926. doi: 10.1038/srep36926 (PMC5109406; doi:10.1038/srep36926)
Supplement: Supplementary Information [file srep36926-s1.pdf]

Fasting and Feeding Signals Control the Oscillatory Expression of *Angptl8* to Modulate Lipid Metabolism

Fabin Dang[1], Rong Wu[1], Pengfei Wang[1], Yuting Wu[1], Md. Shofiul Azam[1], Qian Xu[2], Yaqiong Chen[1] and Yi Liu[1]*

[1] Key Laboratory of Nutrition and Metabolism, Institute for Nutritional Sciences, Shanghai Institutes for Biological Sciences, University of Chinese Academy of Sciences, Chinese Academy of Sciences, 320 Yueyang Road Shanghai, 200031, China

[2] Department of Endocrinology, The First Affiliated Hospital of Harbin Medical University, No. 23 Youzheng Street, NanGang District, Harbin, 150001, China

*To whom correspondence should be addressed:

Yi Liu

liuyi@sibs.ac.cn

Phone: 086-21-54920762

FAX: 086-21-54920291

Table 1. Primers used for cloning	
Angptl8-P3K-F	CCGCTCGAGTGGCAGGCAGAGAGGACTGACTC
Angptl8-P3K-R	CCCAAGCTTGTTTCGTGGGTGTGGTTCCC
Angptl8-P2K-F	CCGCTCGAGGTCACTGCACTGACCGGCAA
Angptl8-P2K-R	CCCAAGCTTGTTTCGTGGGTGTGGTTCCC
Angptl8-P1.8K-F	CCGCTCGAGCGGAGGACCCTTTAGAGGGCTG
Angptl8-P1.8K-R	CCCAAGCTTGTTTCGTGGGTGTGGTTCCC
Angptl8-P1.6K-F	CCGCTCGAGCATCCTGGTCTACATAGCAAG
Angptl8-P1.6K-R	CCCAAGCTTGTTTCGTGGGTGTGGTTCCC
Angptl8-P1.4K-F	CCGCTCGAGCACACTAGGCAAGTACTCTACC
Angptl8-P1.4K-R	CCCAAGCTTGTTTCGTGGGTGTGGTTCCC
Angptl8-P1.2K-F	CCGCTCGAGCATAAGGTGGTCCCACCAGTAG
Angptl8-P1.2K-R	CCCAAGCTTTGACAGTTTCGTGGGTGTGGTT
Angptl8-P1.0K-F	CCGCTCGAGAGGGTCTGTCTTTCAGATAC
Angptl8-P1.0K-R	CCCAAGCTTTGACAGTTTCGTGGGTGTGGTT
Angptl8-3UTR-F	GCTCTAGAGACTACCTGGATGCCACCGAGGAC
Angptl8-3UTR-R	ACGCGTCGACTAGATTGCCTCTGTTCTCTT
Angptl8-delte-LXRE-1-F	ACCGGCCATGTACTGGATCCGC
Angptl8-delte-LXRE-1-R	GCCAGGCCTTGCCGGTCAGTGC
Angptl8-delte-LXRE-2-F	ACAGAGCCCTGGCGACTGCT
Angptl8-delte-LXRE-2-R	GCTGTGCGGATCCAGTACATGG
Angptl8-delte-LXRE-3-F	GAAAAGGAGGGGTGGAGGGACGGAGGACCCTTTA
Angptl8-delte-LXRE-3-R	GCTCTTCGTGTACCCACACAGCCTCAATTTCAG
Angptl8-delte-LXRE-4-F	GCCAAAGCTACATAGGGAAATC
Angptl8-delte-LXRE-4-R	CTGACTCTCAGCACCCATCTG
Angptl8-delte-ngRE-F	TTATCTATAGGGTCTGTCTTTC
Angptl8-delte-ngRE-R	CCAATAAGGTGGTAGTGTGGG
Angptl8-F	ATAAGAATGCGGCCGCATGGCTGTGCTTGCTCTCTGCCT
Angptl8-R	CGGGATCCGGCTGGGAGGGCTGCTGTGTGGA
Angptl8-delte-SP-F	ATAAGAATGCGGCCGCGGTCCAGAGCCAGCTCAATATG
Angptl8-delte-SP-R	CGGGATCCGGCTGGGAGGGCTGCTGTGTGGA
Gr-F	ATAAGAATGCGGCCGCgaATGGACTCCAAAGAATCCTTAGC
Gr-R	GCTCTAGATCATTTCTGATGAAACAGAAGC
Lxra-F	ATAAGAATGCGGCCGCgaATGTCCTTGTGGCTGGAGGCCTCAAT
Lxra-R	GCTCTAGATCACTCGTGGACATCCCAGATCTCAG

Table 2. Primers used for qPCR	
mAngptl8-F	CACTGTACGGAGACTACAAGTGC
mAngptl8-R	GTGGCTCTGCTTATCAGCTCG
mAngptl3-F	CCAGAACACCCAGAAGTAACT
mAngptl3-R	TCTGTGGGTTCTTGAATACTAGTC
mAngptl4-F	AAGATGCACAGCATCACAGG
mAngptl4-R	ATGGATGGGAAATTGGAGC
mGr-F	AGCTCCCCCTGGTAGAGAC
mGr-R	GGTGAAGACGCAGAAACCTTG
mNr1d1-F	TCCCCAAGAGAGAGAAGCAA
mNr1d1-F	CTGAGAGAAGCCCACCAAAG
mSrebp-1c-F	GGAGCCATGGATTGCACATT
mSrebp-1c-R	GCTTCCAGAGAGGAGCCCAG
mFasn-F	GCTGCGGAAACTTCAGGAAAT
mFasn-R	AGAGACGTGTCACTCCTGGACTT
mAcc-F	TGACAGACTGATCGCAGAGAAAG
mAcc-R	TGGAGAGCCCCACACACA
mTgh-F	GGAGGGCAGGTGCTCTCA
mTgh-R	GCCTTCAGCGAGTGGATAGC
mDgat1-F	GGATCTGAGGTGCCATCGT
mDgat1-R	CCACCAGGATGCCATACTTG
mDgat2-F	GGCTACGTTGGCTGGTAACTT
mDgat2-R	TTCAGGGTGACTGCGTTCTT
mApoCIII-F	GGCTGGATGGACAATCACTT
mApoCIII-R	TGGTTGGTCCTCAGGGTTAG
mAbca1-F	TTGGATGGATTAGATTGGAC
mAbca1-R	ATGCCTGTGAACACGATG
mFatp2-F	TCGTGGGACTGGTAGATTTTG
mFatp2-R	CGCGATGTGTTGAAAGAGTTT
mCd36-F	GATGACGTGGCAAAGAACAG
mCd36-R	TCCTCGGGGTCCTGAGTTAT
mFatp4-qF	GTGAGATGGCCTCAGCTATC
mFatp4-qR	GAAGAGGGTCCAGATGCTCT
mAtgl-qF	CTTCCTCGGGGTCTACCACA
mAtgl-qR	GCCTCCTTGGACACCTCAATAA
mHsl-qF	TCAACCGACCAGGAGTGCT
mHsl-qR	CTCGTTGCGTTTGTAGTGCTC
mMgl-qF	CGGACTTCCAAGTTTTTGTCAGA
mMgl-qR	GCAGCCACTAGGATGGAGATG
